# Supplementary material for: A proteomics landscape of circadian clock in mouse liver
Source: Nat Commun. 2018 Apr 19;9:1553. doi: 10.1038/s41467-018-03898-2 (PMC5908788; doi:10.1038/s41467-018-03898-2)
Supplement: Supplementary file 1 — Supplementary Information [file 41467_2018_3898_MOESM1_ESM.pdf]

A proteomics landscape of circadian clock in mouse liver

Wang et al.

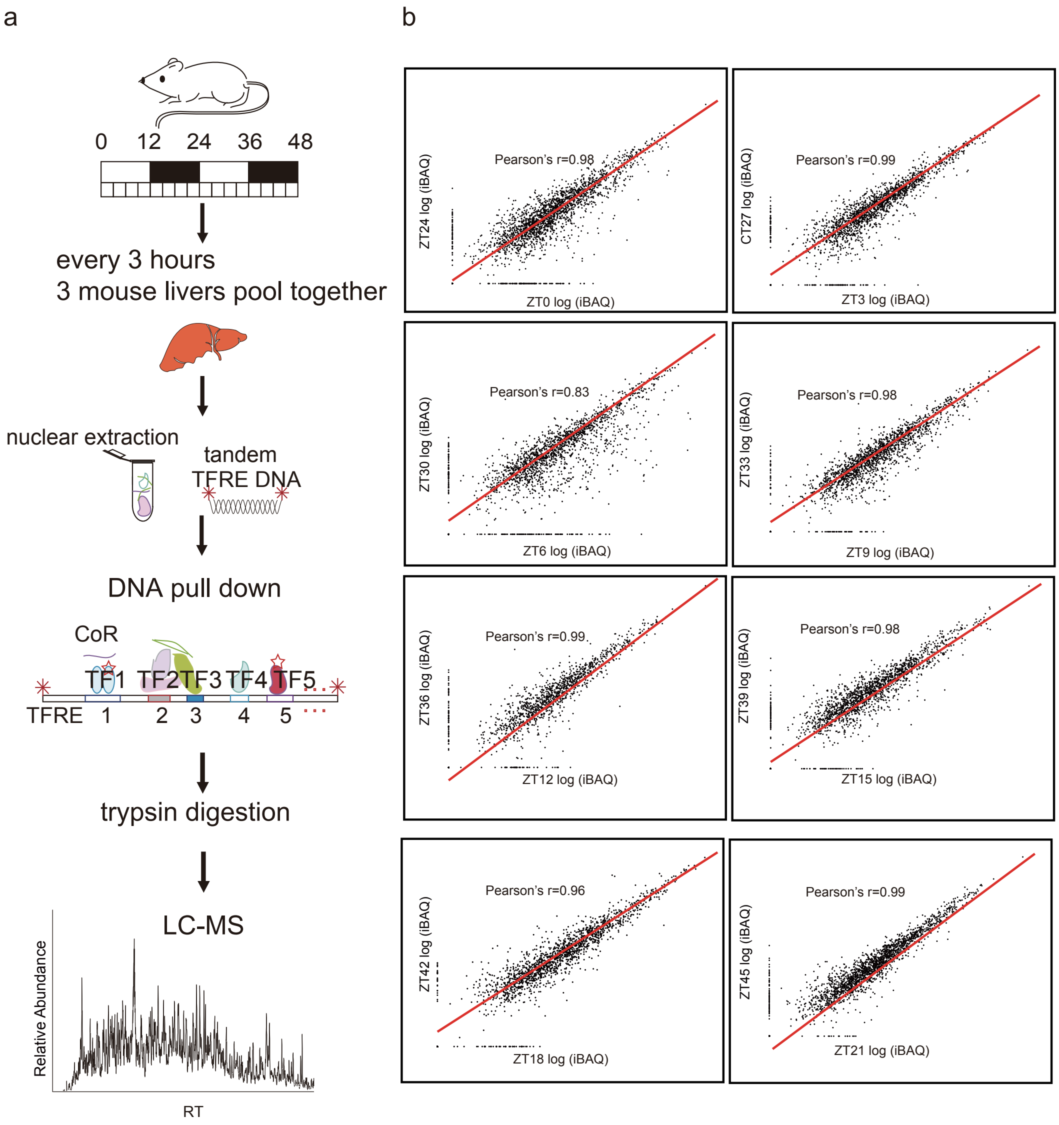

Supplementary Fig. 1 Deep TF DNA-binding Activity Profiling (DBAP) of circadian clock in mouse liver. (a) Systematic workflow of the experimental strategy to profile the diurnal rhythmic TF DNA-binding activity in mouse liver. (b) The correlation coefficient between samples at the replicate time point during 2 consecutive cycles. Red line presents linear regression of the data and Pearson *r* was calculated for each pair of the samples. Related to **Fig. 1**.

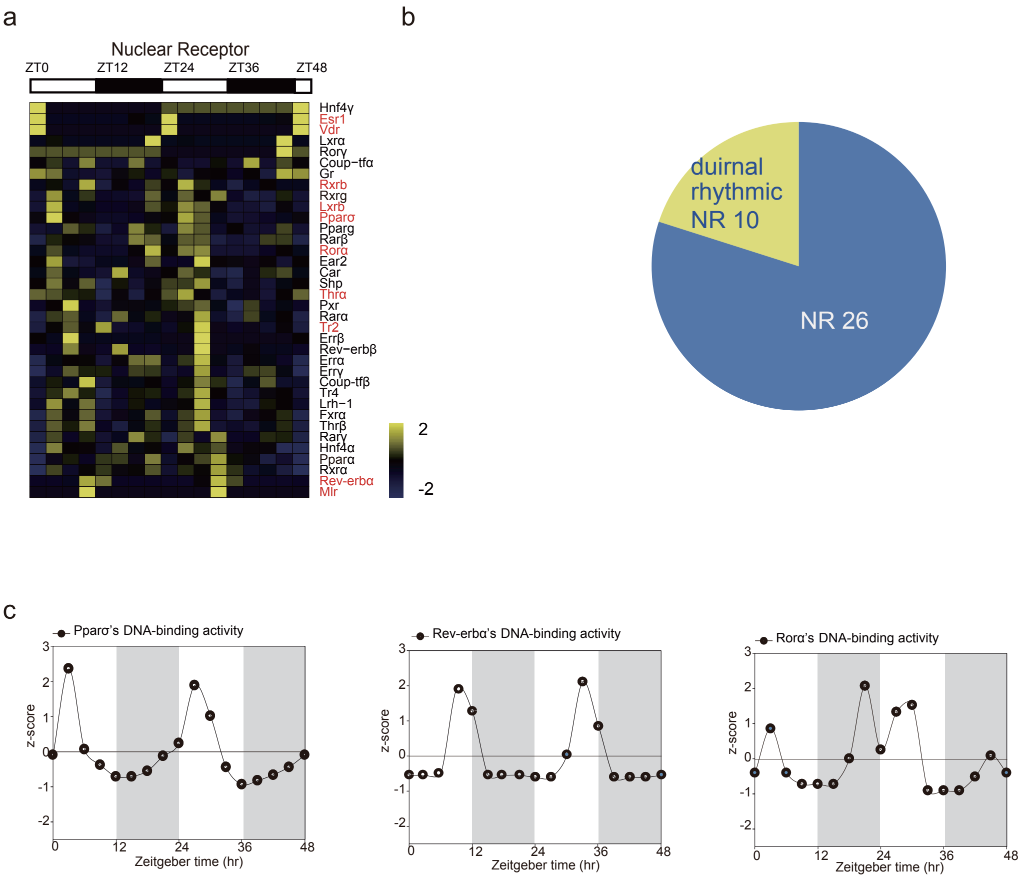

Supplementary Fig. 2 The circadian rhythm of Nuclear Receptors super family. (a). Hierarchical clustering of the proteins ordered by the phase of the oscillation. Values for each protein at all analyzed samples (columns) are color code based on the intensities, low (blue) and high (yellow) z-scored normalized iBAQ. The upper white to black bar indicates the 2 days' cycle. Daytime is shown in white, while nighttime is shown in black. NRs colored in red are the diurnal rhythmic ones (JTK_CYCLE, p value<0.1). (b) Pie graph shows the distribution of rhythmic NRs. (c) Temporal levels of the abundance of DNA-binding activity of Pparσ, Rev-erbα and Rorα in 2 consecutive cycles. X axis represents the sampled time points, Y axis represents the relative abundance of DNA-binding activity (normalized z-scored iBAQ) of each protein.

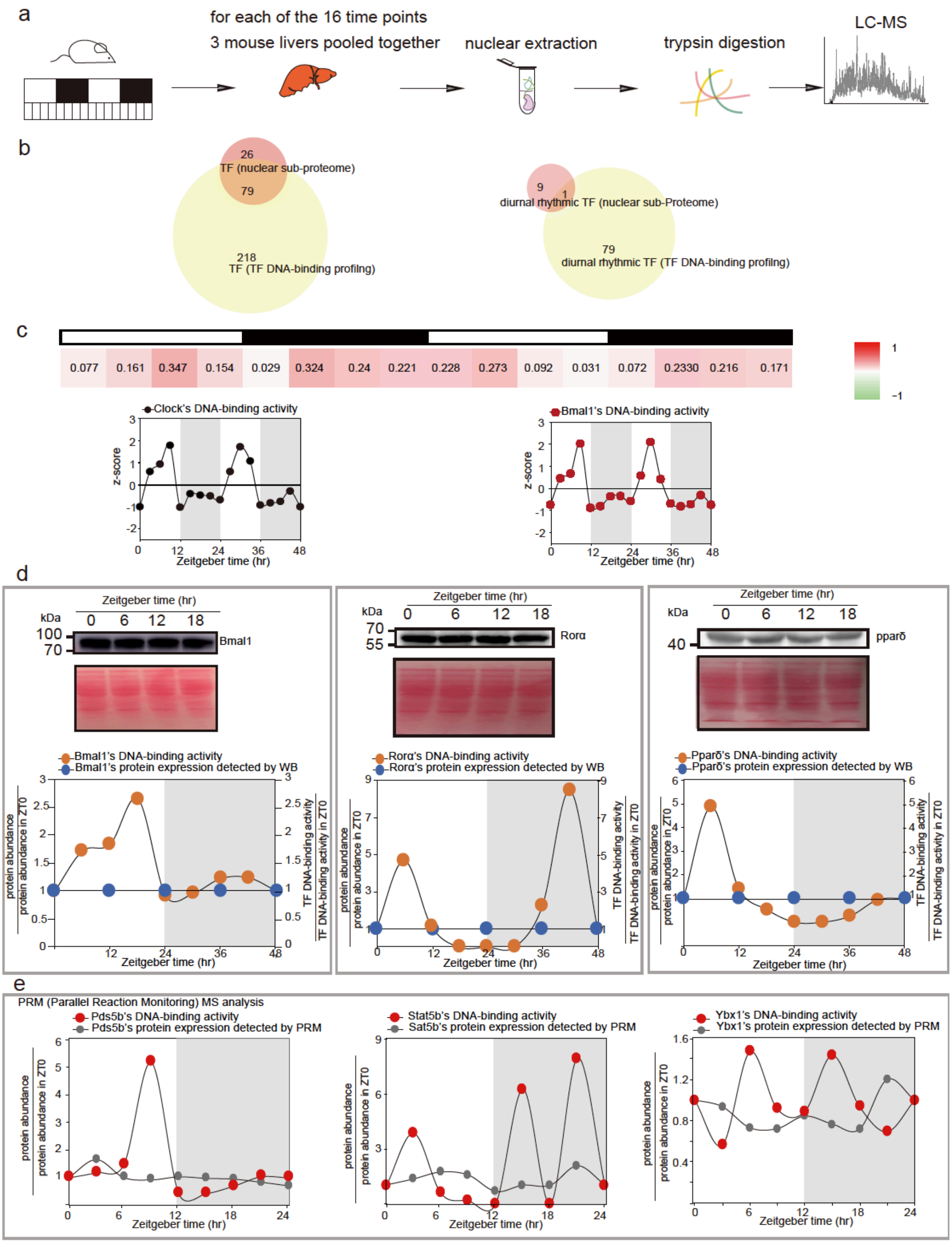

Supplementary Fig. 3 Comparison between TF DNA-binding activity profiling and liver nuclear proteome of the circadian clock in mouse liver. (a) Systematic workflow of the experimental strategy to profile the nuclear sub-proteome of diurnal rhythm in mouse liver. (b) Venn diagram shows the overlap between TFs detected by the nuclear sub-proteome, and by the DNA-binding activity profiling. (c) Heat map of correlation between nuclear proteins' expression and nuclear proteins' DNA-binding activity at each time point. Values for each pair of comparison are color code based on the correlation, low (white) and high (red). The upper white to black bar indicates the 2 days' cycle. Daytime is shown in white, while nighttime is shown in black. The example of temporal abundance of DNA-binding activity of Bmal1 and

Clock. X axis represents the sampled time points, Y axis represents the relative abundance of DNA-binding activity (normalized z-scored iBAQ) of each protein. (d, e) Comparison between protein's abundance in the whole liver proteome and its DNA-binding activity. The temporal protein abundance of Bmal1, Rorα, Pparδ, conformed by Western Blotting (WB) in (d). X axis represents the sampled time points, Y axis represents ratio against protein's abundance of ZT0. The graph on the top presents the band blots of western blots, with loading control. The temporal protein abundance of Pds5b, Stat5b, Ybx1, conformed by PRM-MS analysis in (e). X axis represents the sampled time points, Y axis represents ratio against protein's abundance of ZT0.

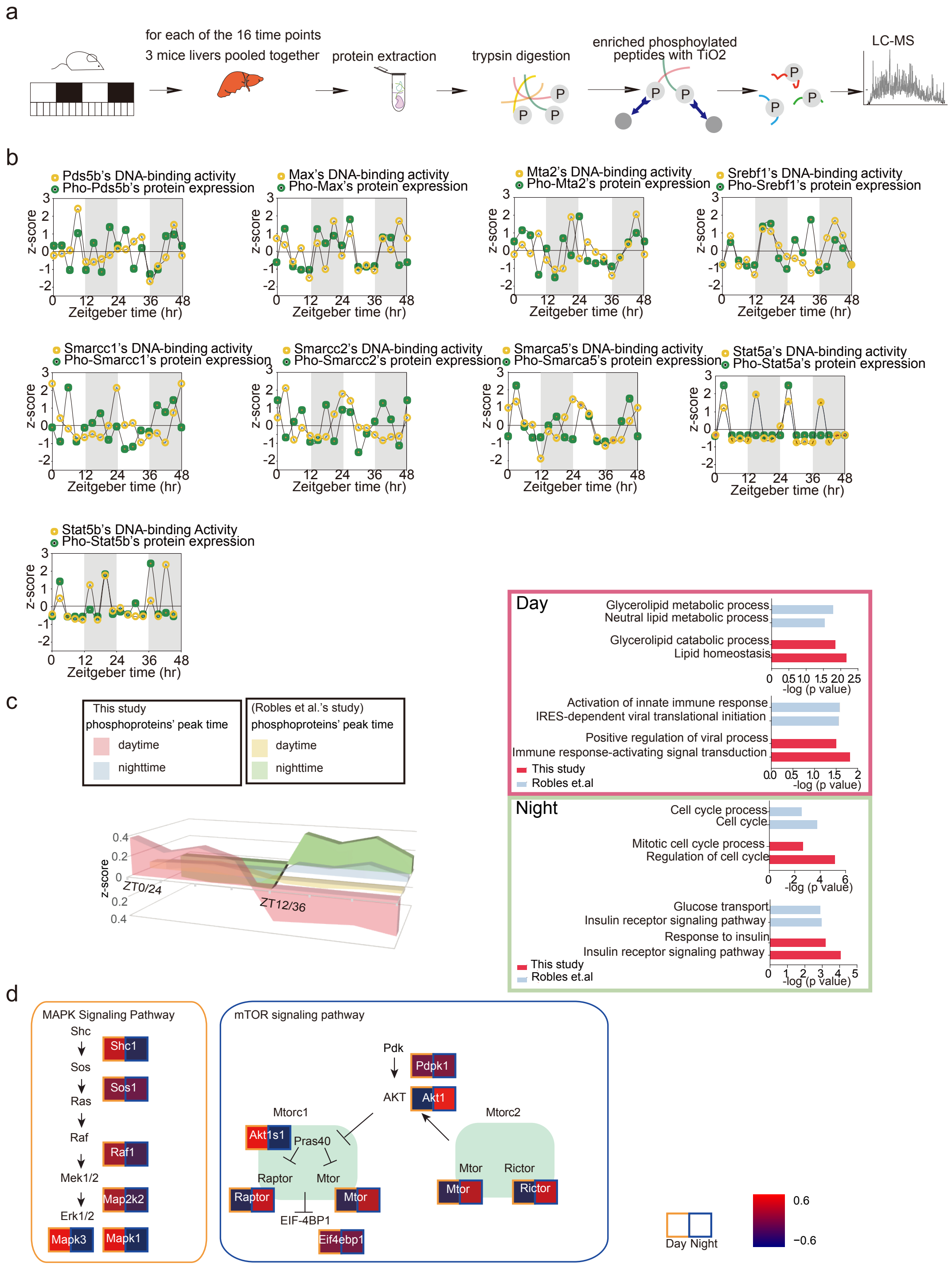

Supplementary Fig. 4 Phosphoproteome of circadian clock in mouse liver. (a) Systematic workflow of the experimental strategy to profile the phosphoproteome of diurnal rhythm in mouse liver. (b) Temporal abundance of DNA-binding activity of TFs and their correlated abundance of their phosphorylated forms. X axis represents the sampled time points, Y axis represents the z-scored abundance. (c) Area graph shows the normalized abundance of phosphoproteins that peaked in different time of the day. Bar plots shows the GO terms enriched by diurnal peaked phosphoproteins. (d) Expression patterns of the pathway specific phosphoproteins. Expression level of each phosphoprotein is color coded based on average of

normalized z-scored iBAQ of the daytime or of nighttime, low (blue) and high (red) z-scored normalized iBAQ. Related to **Fig. 3**.

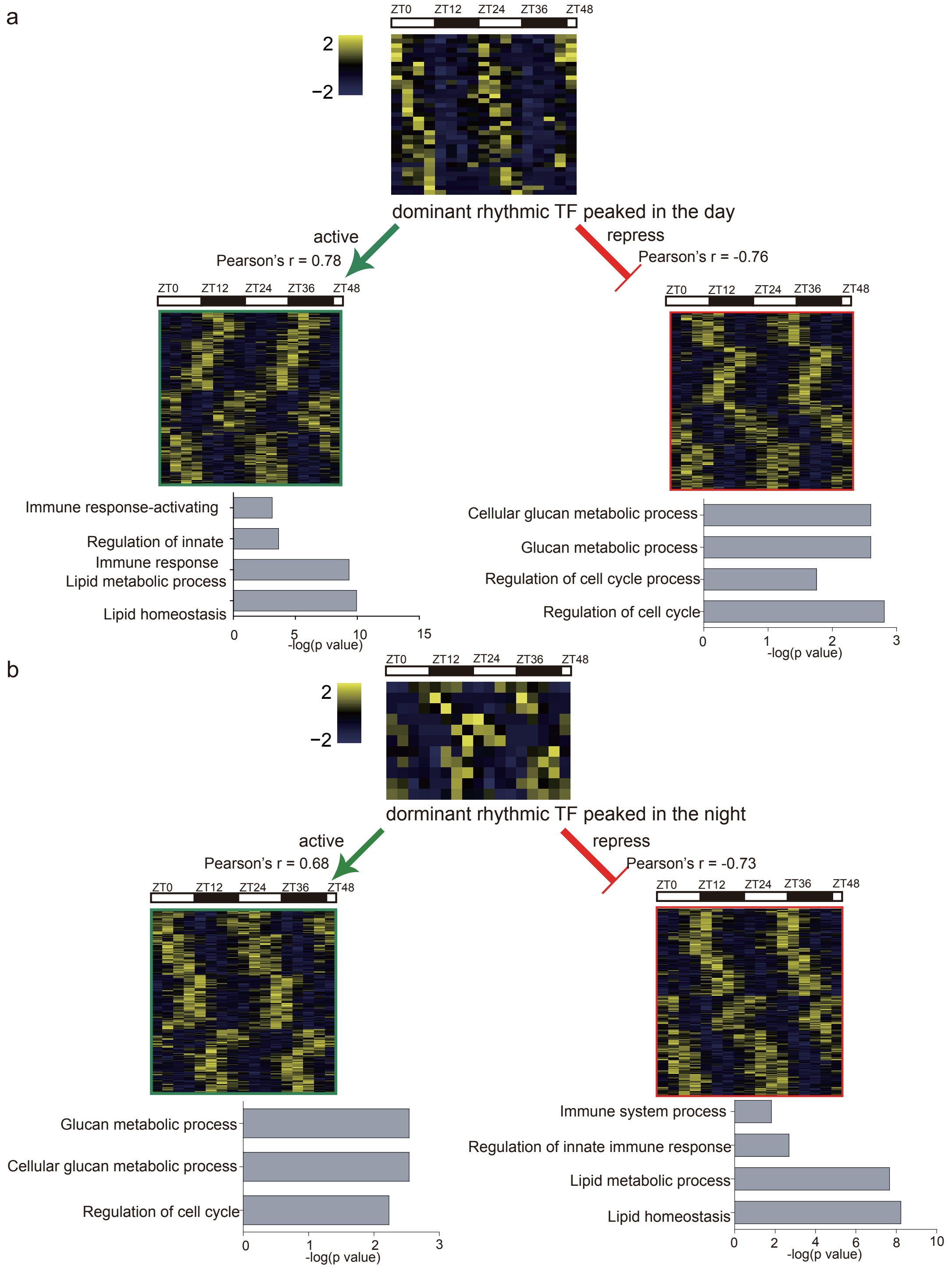

Supplementary Fig. 5 The "activation" and "repression" effects of the dominant rhythmic TFs (DR-TFs) to their target genes. (a, b) Diurnal peaked DR-TFs (a shows daytime peaked DR-TFs, b shows nighttime peaked DR-TFs) and their activated and repressed TGs are shown by hierarchical clustering heat maps. Proteins ordered by the phase of the oscillation. Values for each protein at all analyzed samples (columns) are color code based on the intensities, low (blue) and high (yellow) z-scored normalized iBAQ. The white to black bar indicates the 2 days' cycle. Daytime is shown in white, while nighttime is shown in black. Bar plots show the GO bioprocesses TGs enriched in. Related to **Fig. 4**.

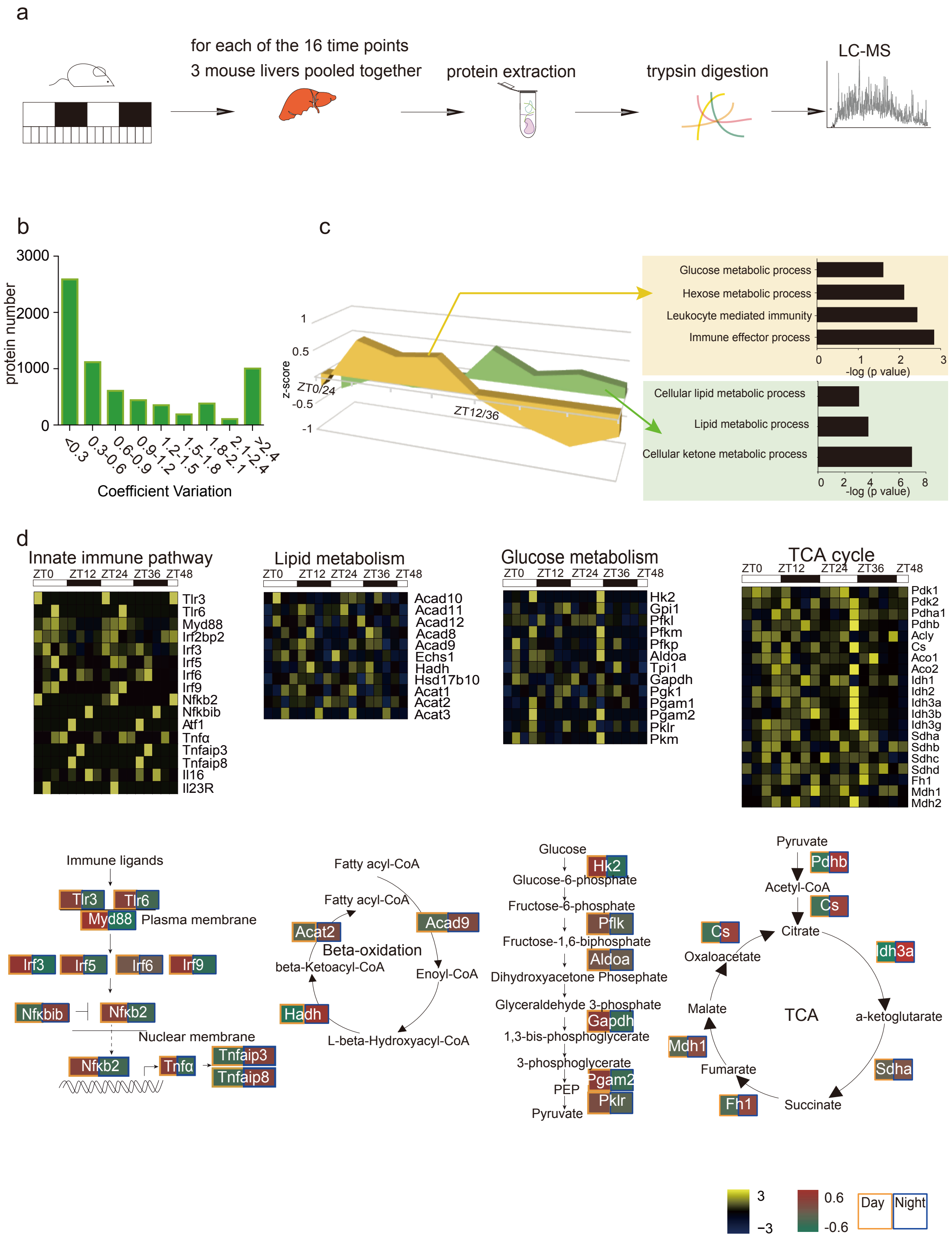

Supplementary Fig. 6 Whole tissue proteome of circadian clock in mouse liver. (a) Systematic workflow of the experimental strategy to profile the proteome of diurnal rhythm in mouse liver. (b) Histogram of the number of proteins classified based on their coefficient variation. (c) Area graph shows the normalized abundance of rhythmic proteins peaked in different time of the day (yellow: daytime green: nighttime). Bar plots shows the GO terms enriched by diurnal peaked proteins. (d) Hierarchical clustering of the cycling proteins ordered by the phase of the oscillation. Values for each protein at all analyzed samples (columns) are color code based on the intensities, low (blue) and high (yellow) z-

scored normalized iBAQ. The upper white to black bar indicates the 2 days' cycle. Daytime is shown in white, while night time is shown in black. Expression patterns of the pathway specific proteins. Expression level of each protein is color code based on their average intensity of the daytime or nighttime, low (green) and high (red) z-scored normalized iBAQ. Related to **Fig. 5.**

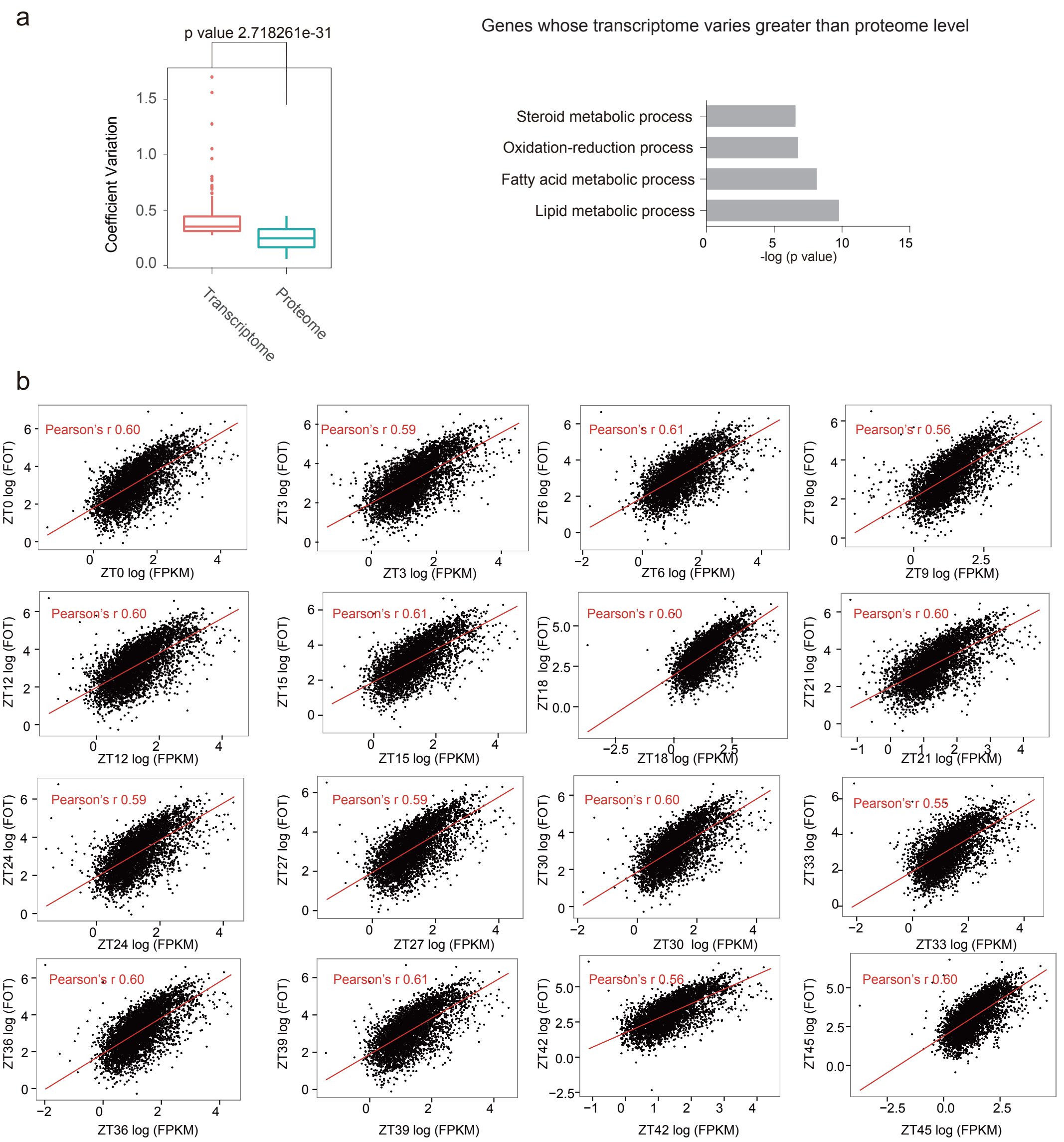

Supplementary Fig. 7 Comparison between diurnal rhythmic transcriptome and proteome (a) The box plot indicates the comparison between coefficient variation of proteome and of transcriptome (Pair tailed student's t test p value<0.05). For the box plot, the bottom and top of the box are the first and third quartiles, and the band inside the box is the median of the coefficient variation of proteome and of transcriptome. The bar plot shows the GO bioprocess enriched by the genes whose coefficiency variation at mRNA level changes greater than their coefficiency variation at protein level. (b) The scatter plots show the correlation coefficient between iBAQ intensities of proteome versus FPKM values of transcriptome. Red line presents linear regression of the data and Pearson r was calculated for each pair of the samples. Related to **Fig. 5.**

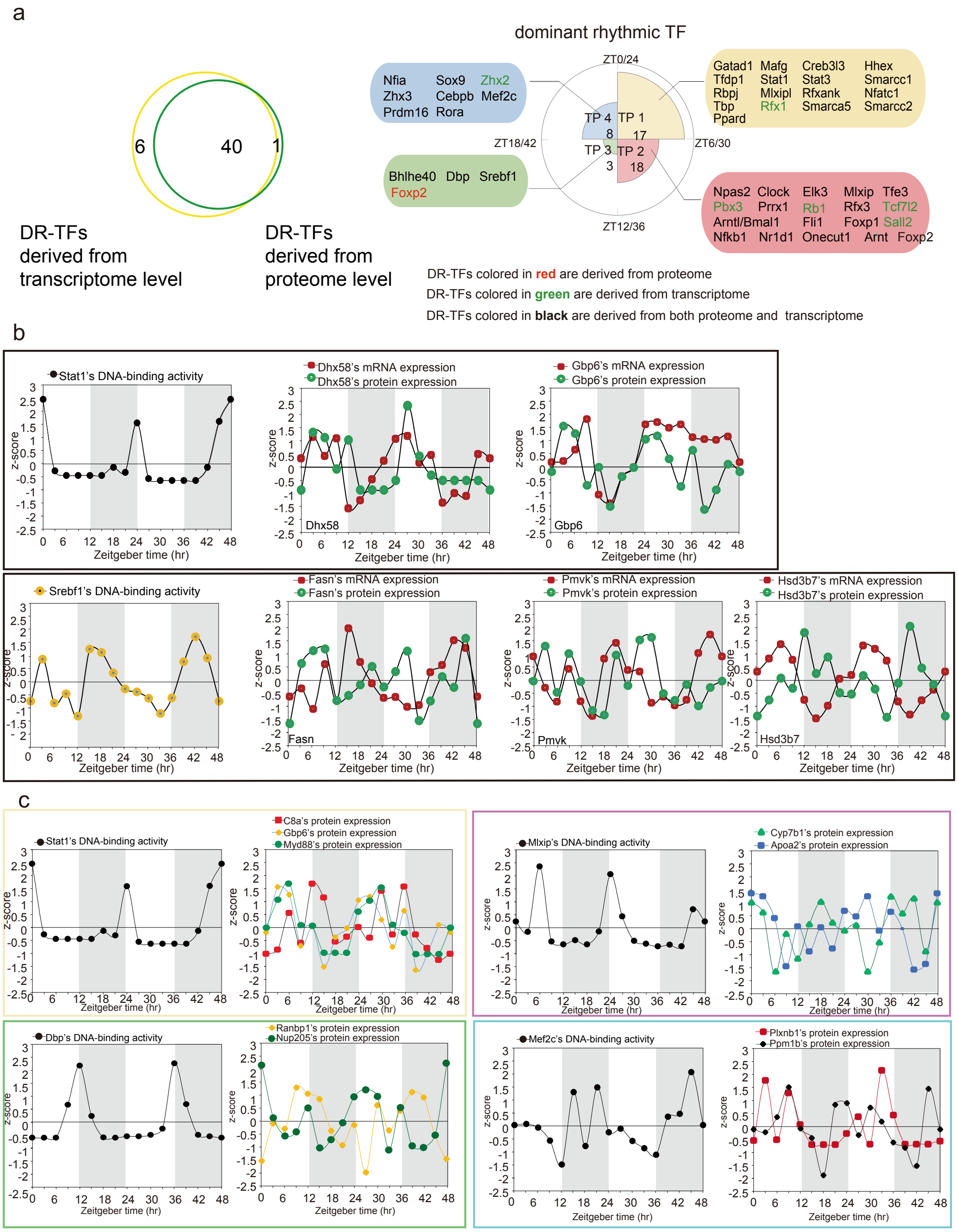

Supplementary Fig. 8 Integration of TF sub-proteome, transcriptome and global proteome. (a) Venn diagram represents overlap of DR-TFs nominated from transcriptome and proteome. The Rose diagram shows the number of transcriptome and proteome nominated DR-TFs of each time phase, TFs were colored based on their peaked phase, (TP1 shows in yellow, TP2 shows in red, TP3 shows in green, TP4 shows in blue). (b) Examples of temporal abundance of DNA-binding activity of DR-TFs, and of

temporal abundance of their TGs at transcript level and protein level. X axis represents the sampled time points, Y axis represents the relative abundance (normalized z-scored iBAQ or z-scored FPKM) of TGs. (c) Examples of temporal abundance of DNA-binding activity of DR-TFs and the temporal abundance of their TGs at protein level, in 2 consecutive cycles. X axis represents the sampled time points, Y axis represents the relative abundance (normalized z-scored iBAQ) of TGs. Related to **Figure 4, 5**.

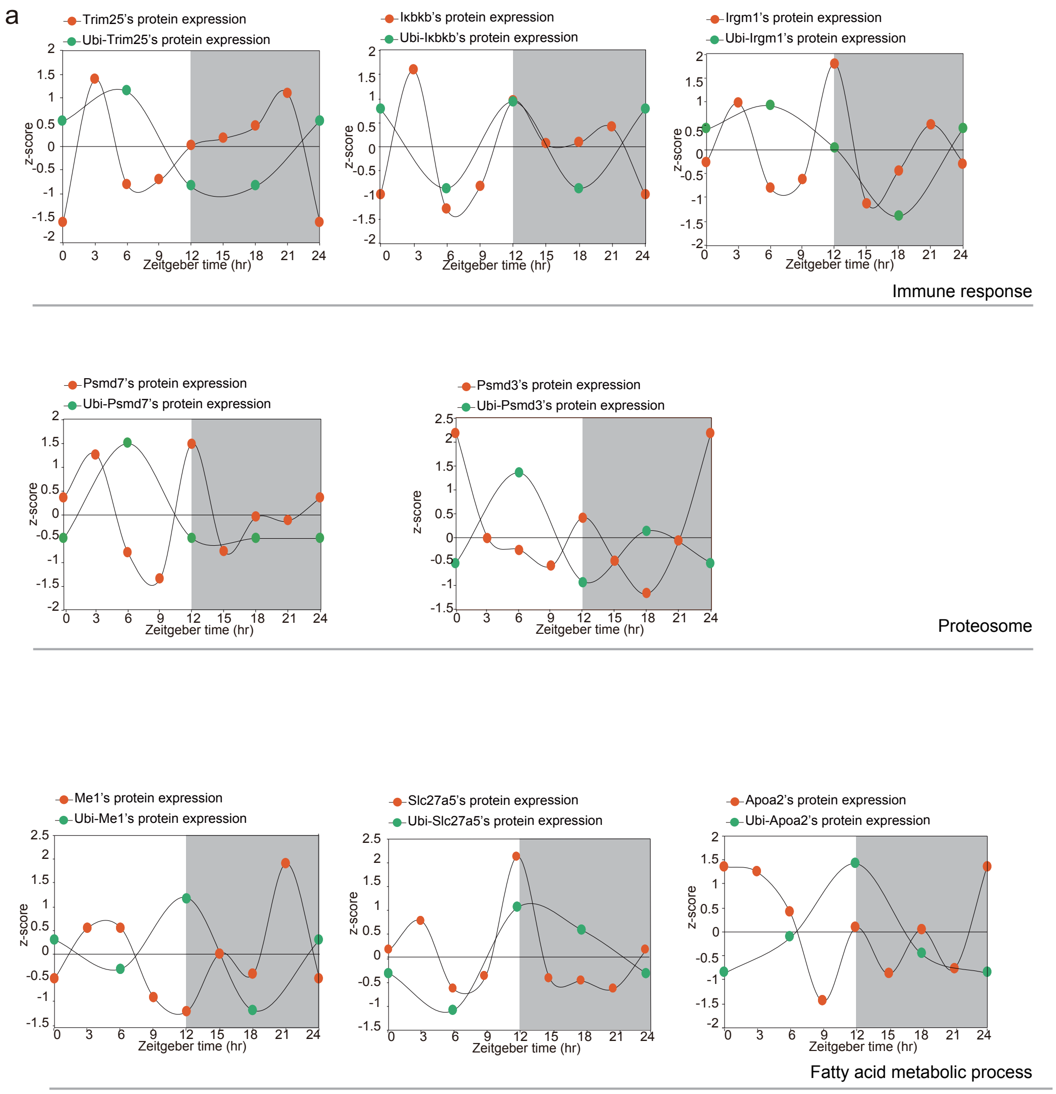

Supplementary Fig. 9 The ubiquitylation pattern of circadian clock in mouse liver. (a) Temporal abundance of proteins and of their ubiquitylated forms. X axis represents the sampled time points, Y axis represents the proteins' abundance (normalized z-scored iBAQ). Related to **Fig. 5**.

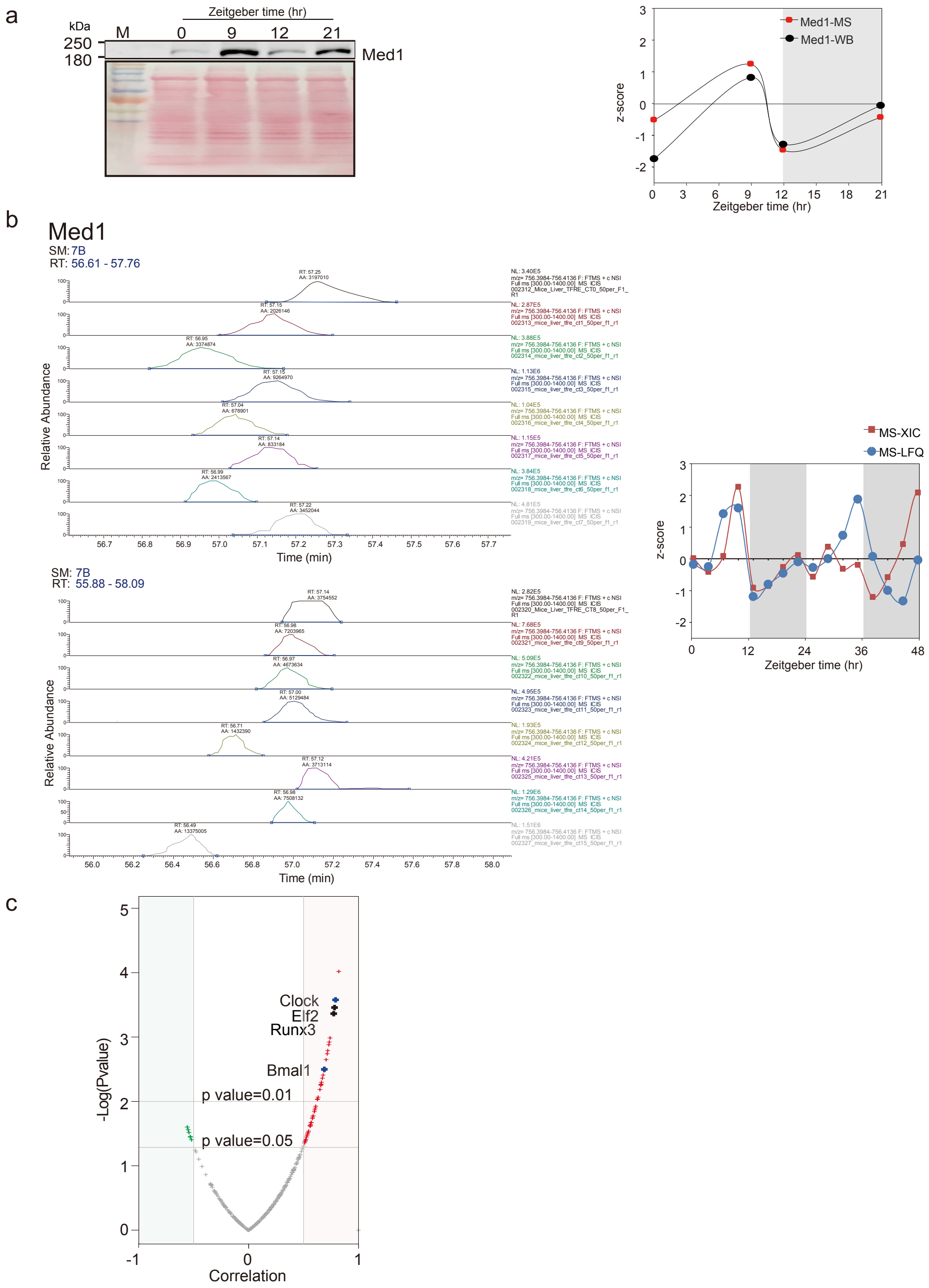

Supplementary Fig. 10 The circadian rhythm of Med complex. (a). The temporal levels of the rhythmic abundance of Med1' DNA-binding activity confirmed by western blotting (WB) analysis. X axis represents the sampled time points, Y axis represents relative abundance (normalized z-scored iBAQ or z-scored densitometry). The graph on the left presents the band blots of western blots, with loading control. (b) Med1's XICs extracted manually from raw files of each ZT point. The temporal levels of

Med1' DNA-binding activity confirmed by the quantification result from manually extracted XICs (red). X axis represents the sampled time points, Y axis represents the relative abundance (normalized z-scored iBAQ). (c) Volcano plot showing rhythmic TFs' correlation with Med1. Related to **Fig. 6**.

a

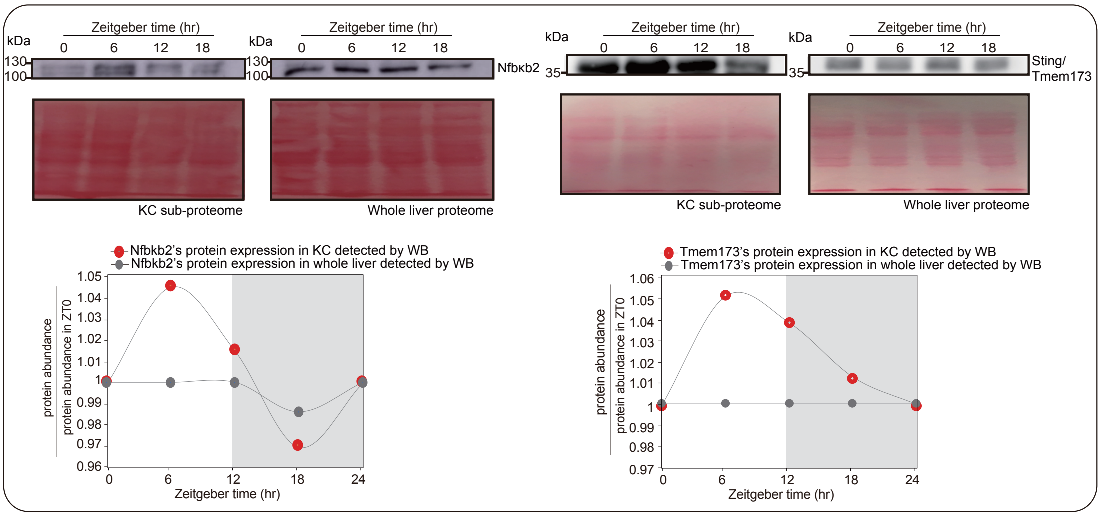

b

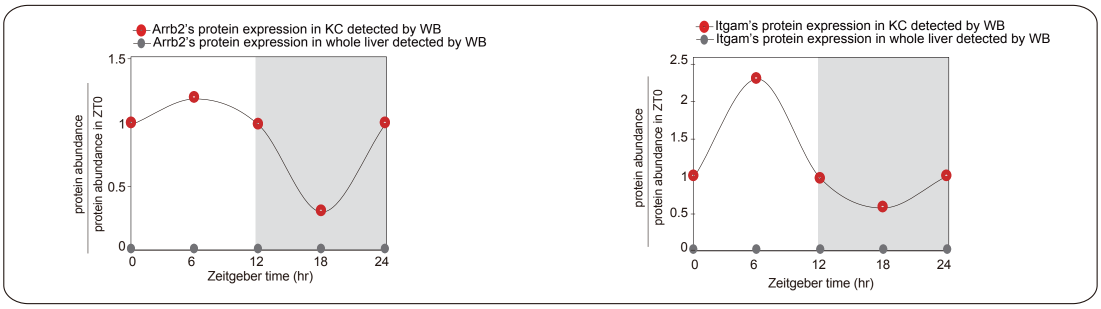

Supplementary Fig. 11 The diurnal rhythm of Kupffer cell proteome. (a) The temporal protein abundance of Nfbκb2, Tmem173 in KCs and in whole liver, conformed by WB (Western Blotting). X axis represents the sampled time points, Y axis represents ratio against protein's abundance of ZT0. The graph on the top presents the band blots of western blotting, with loading control. (b) The temporal protein abundance of Arrb2, Itgam in KCs and in whole liver, conformed by DDA MS analysis. X axis represents the sampled time points, Y axis represents ratio against protein's abundance of ZT0. Related to **Fig. 6**.

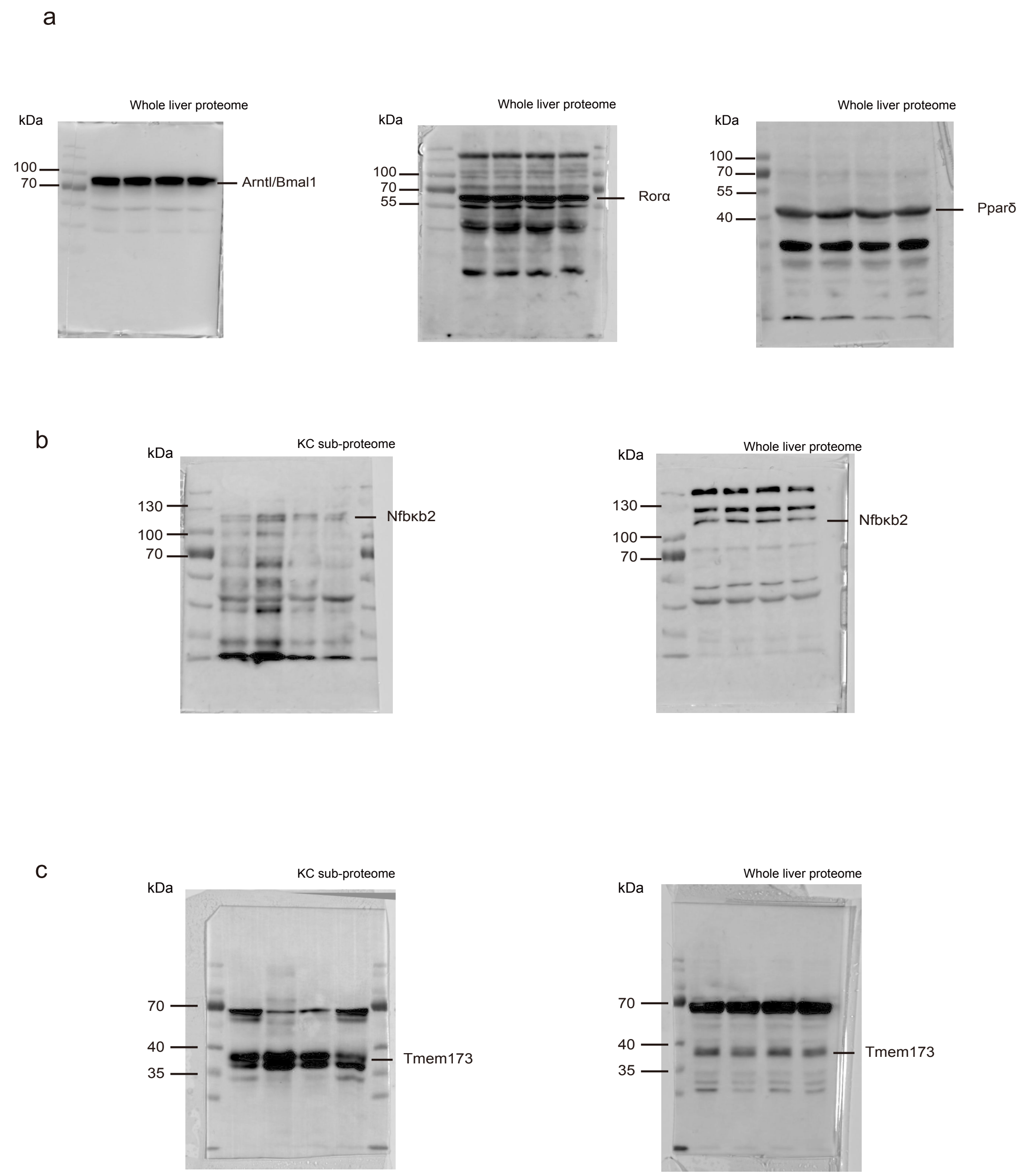

Supplementary Fig. 12 The uncropped western blots of Arntl/Bmal1, Rorα, Pparδ, Nfκb2, and Tmem173. (a) The uncropped western blots of Arntl/Bmal1, Rorα, Pparδ. (b) The uncropped western blots of Nfκb2. (c) The uncropped western blots of Tmem173.
