# Supplementary material for: A proteomics landscape of circadian clock in mouse liver
Source: Nat Commun. 2018 Apr 19;9:1553. doi: 10.1038/s41467-018-03898-2 (PMC5908788; doi:10.1038/s41467-018-03898-2)
Supplement: Supplementary file 3 — Description of Additional Supplementary Files [file 41467_2018_3898_MOESM3_ESM.pdf]

Description of Additional Supplementary Files

File Name: Supplementary Data 1
Description: Matrix of deep TFs DNA-binding activity profiling (DBAP) of diurnal rhythm in mouse liver.
Sheet 1-3 represent matrix of all detected TFs, TCs, and DBPs DNA-binding activity profiling.
Sheet 4-6 represent matrix of diurnal rhythmic TFs, TCs and DBP DNA-binding activity profiling (JTK_CYCLE, p value<0.1).
Sheet 7 represents matrix of DNA-binding activity profiling of Nuclear receptor family.
Sheet 8 represents matrix of DNA-binding activity profiling of Med complex.
Sheet 9 represents TFs whose DNA-binding activity positively correlated with Med1 (Pearson's r>0.5, p value<0.05).

File Name: Supplementary Data 2
Description: GO bioprocess enriched by phase-specific TFs and by diurnal rhythmic phasespecific TFs.
Sheet 1-4 represent GO bioprocess enriched by phase-specific TFs and by diurnal rhythmic phasespecific TFs.

File Name: Supplementary Data 3
Description: Matrix of mouse liver nuclear sub-proteome during the circadian cycle.
Sheet 1 represents matrix of all 4,038 proteins identified in nuclear sub-proteome of mouse liver.
Sheet 2 represents diurnal rhythmic proteins identified in nuclear sub-proteome of mouse liver (JTK_CYCLE, p value<0.1).
Sheet 3 represents the TFs that detected in nuclear sub-proteome.

File Name: Supplementary Data 4
Description: Matrix of mouse liver phosphoproteome during the circadian cycle.
Sheet 1 represents phosphopeptides that were identified in phosphoproteome.
Sheet 2 represents the matrix of the 1,657 phosphoproteins that were identified in phosphoproteome.
Sheet 3 represents the matrix of diurnal rhythmic phosphoproteins (JTK_CYCLE, p value<0.1).
Sheet 4 represents the matrix of phosphorylated TFs.

File Name: Supplementary Data 5
Description: GO enrichment analysis of the phosphoproteins.
Sheet1,2 shows the GO enrichment of phosphoproteins that peaked in the daytime, or peaked at nighttime (phosphoproteins detected in this study).
Sheet3,4 shows the GO enrichment of phosphoproteins that peaked in the daytime, or peak at nighttime (phosphoproteins detected in Roble et al.'s study).

File Name: Supplementary Data 6
Description: Matrix of mouse liver transcriptome during the circadian cycle.
Sheet 1 represents the matrix of 11,120 transcripts of diurnal rhythm in mouse liver.
Sheet 2 represents the matrix of 2,349 diurnal rhythmic transcripts (JTK_CYCLE, p value<0.05).

File Name: Supplementary Data 7
Description: Matrix of GO bioprocess enriched by the transcriptome.
Sheet 1,2 represent GO bioprocess enriched by the transcripts that peaked in the daytime, or peaked at nighttime.
Sheet 3,4 represent GO bioprocess enriched by the diurnal rhythmic transcripts that peaked in the daytime, or peaked at nighttime.

File Name: Supplementary Data 8
Description: Matrix of dominant diurnal rhythmic TFs.
Sheet 1,2 showing the dominant diurnal rhythmic TFs derived from transcriptome and proteome level.
Sheet 3,4 showing the expression of dominant diurnal rhythmic TFs' downstream TGs on transcriptome and proteome level.
Sheet 5 showing the combination of dominant diurnal rhythmic TFs derived from proteome and transcriptome.

File Name: Supplementary Data 9
Description: Matrix of whole liver proteome during the circadian cycle.
Sheet 1 represents matrix of total 6,780 proteins identified in the whole tissue proteome during circadian cycle in mouse liver.
Sheet 2 represents 575 diurnal rhythmic proteins identified in whole liver proteome during circadian cycle (JTK_CYCLE, p value<0.1).

File Name: Supplementary Data 10
Description: The GO bioprocess enriched by the proteome and transcriptome.
Sheet 1 represents the GO bioprocess that enriched by the rhythmically expressed transcripts and proteins.
Sheet 2 represents the GO bioprocess that enriched by genes that shows rhythmicity at transcripts level but not protein level.
Sheet 3 represents GO bioprocess that enriched by genes that shows rhythmicity at protein level but not transcript level.

File Name: Supplementary Data 11
Description: The "activation" and "repression" effects of the DR-TFs to their downstream genes.
Sheet 1-3 represent daytime peaked DR-TFs and the transcripts "activated" or "repressed" by them.
Sheet 4-6 represent night peaked DR-TFs and the transcripts "activated" or "repressed" by them.
Sheet 7 represents the GO bioprocess enrich by DR-TFs' target genes.

File Name: Supplementary Data 12
Description: Matrix of Ubiquitylation pattern of diurnal rhythm in mouse liver.
Sheet 1 the matrix of 3,424 ubiquitylated peptides.
Sheet 2 represents the matrix of the 1,144 Ubiquitylated proteins.

File Name: Supplementary Data 13
Description: Matrix of Kupffer cell sub-proteome during the circadian cycle.

File Name: Supplementary Data 14
Description: Matrix of peptides analyzed by parallel reaction monitoring (PRM).
